# Occlusion of the common femoral artery by cement after total hip arthroplasty: a case report

**DOI:** 10.1186/1752-1947-3-86

**Published:** 2009-10-30

**Authors:** Mikel L Reilingh, Koen J Hartemink, Arjan WJ Hoksbergen, Rachid Saouti

**Affiliations:** 1 Department of Orthopaedic Surgery, VU Medical Center, De Boelelaan 1117, 1081 HV Amsterdam, the Netherlands; 2 Department of Vascular Surgery, VU Medical Center, De Boelelaan 1117, 1081 HV Amsterdam, the Netherlands

## Abstract

**Introduction:**

The incidence of vascular injuries after total hip arthroplasty is extremely low. In this report we describe an unusual injury to the common femoral artery.

**Case presentation:**

A 59-year-old Caucasian woman presented with rest pain, numbness and cramps in the operated limb after hip replacement. Cement leakage under the transverse ligament had caused occlusion of the common femoral artery necessitating vascular reconstruction. She had a good functional recovery at follow-up.

**Conclusion:**

To the best of our knowledge, this is the first well-documented case reporting this pathomechanism of vascular lesion to the femoral artery. This case report highlights the potential risk of such a limb-threatening complication, and awareness should lead to prevention by meticulous surgical technique (correct technique of pressurization) or to early detection of the lesion.

## Introduction

Total hip arthroplasty (THA) is a successful procedure with a satisfactory outcome in patients with coxarthrosis. Vascular injuries are a rare complication after orthopedic surgery of the hip. The complication rate is reported to be approximately 0.2-0.3% [1-4]. The most commonly injured vessels are the external iliac artery and vein and the common femoral artery [5,6]. Vascular complications are usually caused by direct trauma due to an osteotome, retractor, screws or powered reamer [7]. Direct injury from cement has been reported due to intrapelvic wall penetration, with thermal injury to the external iliac artery causing occlusion by thrombosis [8-10].

An unusual pattern of injury to the common femoral artery after THA is presented. Cement leakage under the transverse ligament caused occlusion of the common femoral artery necessitating vascular reconstruction.

## Case presentation

**Figure 1:**
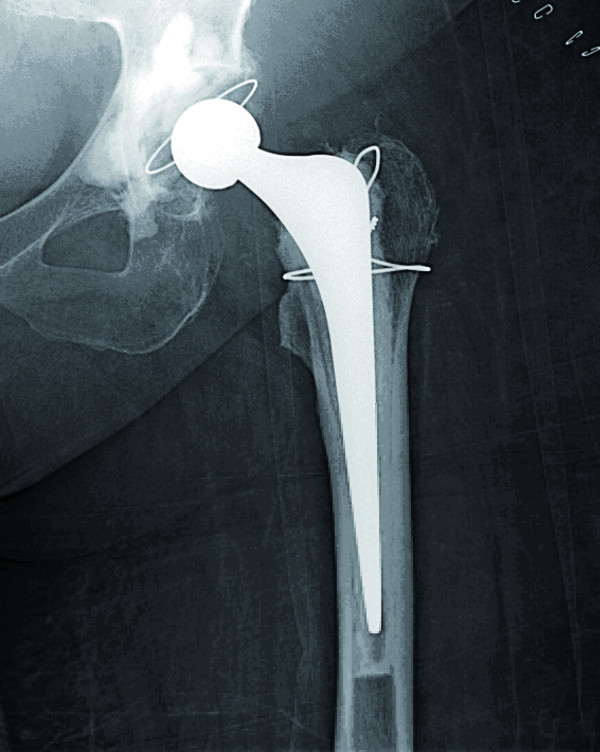
**Anteroposterior radiograph showing the cemented total hip prosthesis with no obvious cement extrusion in the pelvis or soft tissue**.

An obese 59-year-old Caucasian female patient with a history of symptomatic osteoarthritis of the left hip received an Exeter total hip arthroplasty (THA) (Stryker^®^) by the posterolateral approach. The acetabulum was prepared with several anchorage holes and low viscosity Simplex P (Stryker^®^) cement was pressurized. Cerclage wiring was used to control a trochanteric fissure. At that time, no further complications were noted. Postoperatively, the patient complained of severe groin pain. Radiographs showed satisfactory positioning of both components and no cement extrusion into the pelvis or soft tissue was observed (Figure 1). Clinical neurologic and vascular examinations were normal except for marked swelling of the leg. Venous ultrasound was carried out and excluded a deep venous thrombosis. Ten days postoperatively, she was able to walk with partial weight bearing and was discharged from hospital.

**Figure 2:**
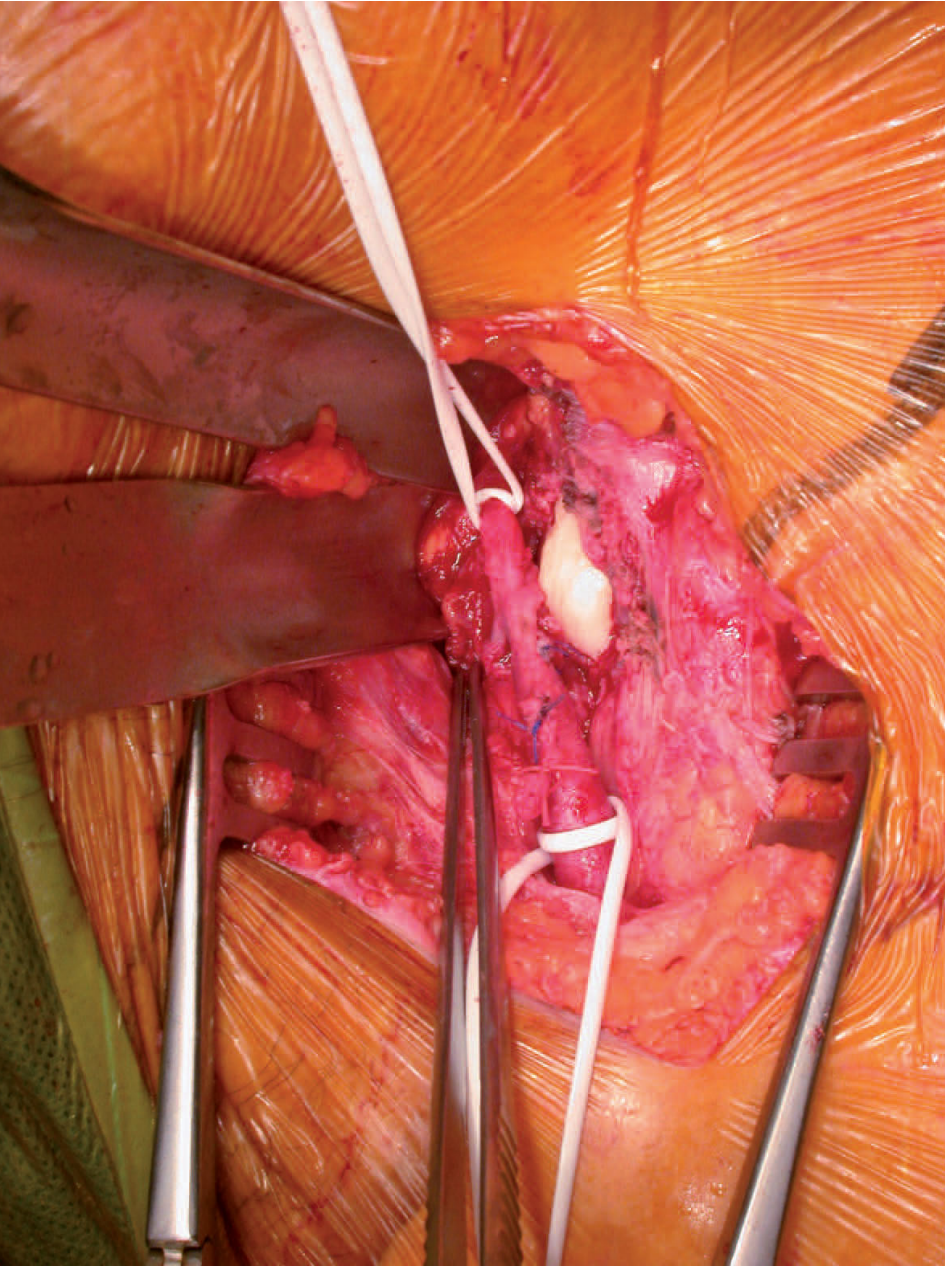
**Lesion of the posterior aspect of the common femoral artery due to cement**.

**Figure 3:**
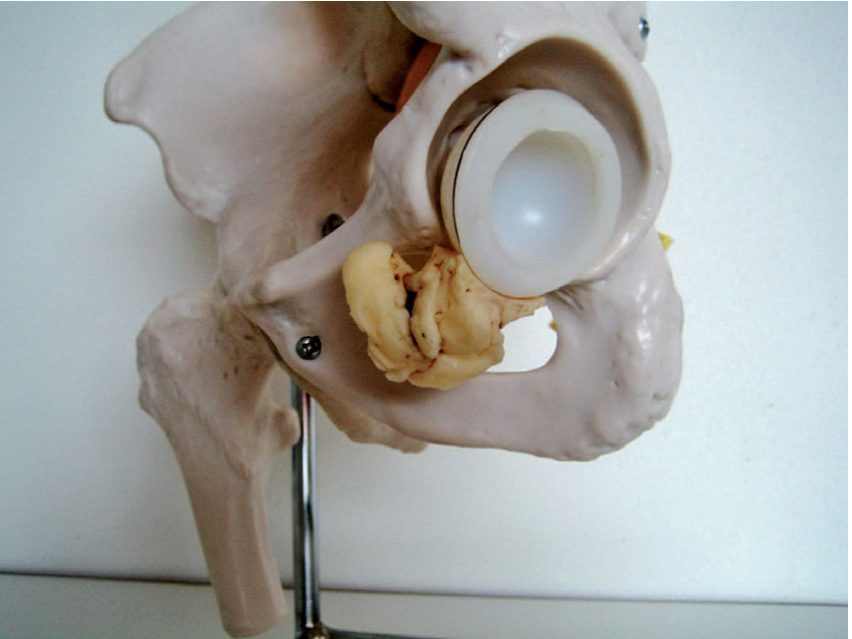
**Reconstruction of the cement leakage under the transverse ligament**.

**Figure 4:**
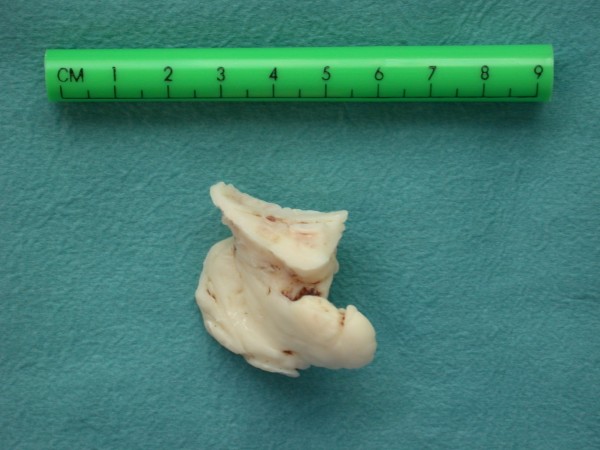
**The unique print of the cemented acetabular cup on the extracted cement mass clearly demonstrates the pathomechanism of leakage under the transverse ligament**.

Two months later, she presented to our outpatient clinic with excruciating rest pain, numbness and cramps in the operated limb. Physical examination showed paleness and peripheral pulselessness of the left leg. The ankle brachial index was 0.4. The patient did not have a medical history of peripheral arterial occlusive disease nor did she have risk factors for atherosclerosis. All peripheral pulsations on the contralateral leg were present and the ankle brachial index of this leg was 1. Arterial duplex examination showed a normal aorto-iliac segment, an occlusion of the left common femoral artery and a normal superficial and deep femoral artery. Inguinal exploration was performed showing a large mass of cement crushing the posterior aspect of the common femoral artery (Figure 2, Figure 3 and Figure 4). The cement was removed with an osteotome. After successful thrombectomy of the common femoral artery and resection of the crushed segment, a Dacron^® ^graft interposition was inserted. The postoperative course was uneventful with complete relief of the symptoms.

## Discussion

Despite the proximity of the pelvic vascular structures to the hip joint, vascular complications after THA are not common. Nevertheless, they can lead to a poor outcome and be potentially limb threatening.

In this case, an unusual pathomechanism of a vascular lesion to the common femoral artery is presented. The print of the cemented acetabular cup on the extracted cement mass confirmed the diagnosis of the vascular surgeon that cement leakage had occurred under the transverse ligament during pressurization of the cup. To the best of our knowledge, this pattern of cement migration causing a vascular lesion has never been reported before.

We theorize that a crush injury of the common femoral artery by cement could have been an early complication, because the patient reported severe postoperative groin pain. The first time the patient presented with vascular complications was 2 months after surgery. During this period, it is probable that the common femoral artery progressively thrombosed and finally occluded. We assume that the direct compression of the cement on the posterior wall of the common femoral artery primarily caused a severe stenosis but not a total occlusion. As the patient was still not able to walk long distances due to her recent hip arthroplasty, she did not complain of intermittent claudication. Gradually, the common femoral artery must have thrombosed completely, most likely accelerated by the repetitive additional trauma during ambulation. At the time of vascular reconstruction, a combination of old and relatively fresh thrombus was found in the common femoral artery, supporting this theory.

The aim of pressurization of cement into the cancellous bone is to increase the osseointegration of cement into the cancellous bone around the acetabulum to produce a more complete cement-bone interface. Adequate generation of pressure is difficult to achieve because of the discontinuity of the acetabulum wall under the transverse ligament, the cotyloid notch. Differently shaped and styled commercially available pressurization devices have been designed to combat the possibility of cement extrusion under the transverse ligament out through the acetabular notch but, to date, there is no accepted gold standard design. We believe that pressurization under direct visual control with transparent pressurizers and with superiorly directed force could probably reduce the cement extrusion anteroinferiorly.

## Conclusion

An unusual presentation of an occlusion of the common femoral artery by cement leakage under the transverse ligament during cemented total hip replacement is reported. Attention should be paid to the technique of pressurization of the cup to avoid this type of complication.

## Abbreviations

THA: total hip arthroplasty.

## Consent

Written informed consent was obtained from the patient for publication of this case report and any accompanying images. A copy of the written consent is available for review by the Editor-in-Chief of this journal.

## Competing interests

The authors declare that they have no competing interests.

## Authors' contributions

MLR conceived the study, participated in its design and coordination and helped to draft the manuscript. KJH conducted the literature review and carried out the review of the patient's medical record in order to collect all of the available information. AWJH and RS revised the article for intellectual content. All of the authors read and approved the final manuscript.
